# Preparative Separation of Phenolic Compounds from *Halimodendron halodendron* by High-Speed Counter-Current Chromatography

**DOI:** 10.3390/molecules15095998

**Published:** 2010-08-31

**Authors:** Jihua Wang, Haifeng Gao, Jianglin Zhao, Qi Wang, Ligang Zhou, Jianguo Han, Zhu Yu, Fuyu Yang

**Affiliations:** 1 College of Agronomy and Biotechnology, China Agricultural University, Beijing 100193, China; 2 College of Animal Science and Technology, China Agricultural University, Beijing 100193, China

**Keywords:** preparative separation, phenolic compounds, *Halimodendron halodendron*, high-speed counter-current chromatography

## Abstract

Three phenolic compounds, *p*-hydroxybenzoic acid (**1**), isorhamnetin-3-*O*-β-D-rutinoside (**2**), and 3,3′-di-*O*-methylquercetin (**5**), along with a phenolic mixture were successfully separated from the ethyl acetate crude extract of *Halimodendron halodendron* by high-speed counter-current chromatography (HSCCC) with chloroform-methanol-water-acetic acid (4:3:2:0.05, v/v) as the two-phase solvent system. The phenolic mixture from HSCCC was further separated by preparative HPLC and purified by Sephadex LH-20 to afford quercetin (**3**) and 3-*O*-methylquercetin (**4**). Seven hundred mg of ethyl acetate crude extract was separated by HSCCC to obtain six fractions which were then analyzed by high performance liquid chromatography (HPLC). The HSCCC separation obtained total of 80 mg of the mixture of quercetin (**3**) and 3-*O*-methylquercetin (**4**) (26.43% and 71.89%, respectively) in fraction 2, 14 mg of 3,3′-di-*O*-methylquercetin (**5**) at 95.14% of purity in fraction 3, 15 mg of *p*-hydroxybenzoic acid (**1**) at 92.83% of purity in fraction 5, 12 mg of isorhamnetin-3-*O*-β-D-rutinoside (**2**) at 97.99% of purity in fraction 6. This is the first time these phenolic compounds have been obtained from *H. halodendron*, and their chemical structures identified by means of physicochemical and spectrometric analysis.

## 1. Introduction

Natural products are often obtained by some conventional separation methods such as silica gel, macroporous resin, Sephadex LH-20 and high-performance liquid chromatography (HPLC), which are usually time and solvent consuming, and involve multiple steps. High-speed counter-current chromatography (HSCCC), a support free liquid-liquid partition chromatographic technique, eliminates the irreversible adsorptive loss of sample onto the solid support. Furthermore, the HSCCC method permits directly introduction of crude samples into the coil tube without additional treatments, so it has been successfully applied to isolate and purify a number of natural products [1,2,3,4,5,6,7,8,9]. *Halimodendron halodendron* (Pall.) Voss, a leguminous species, is mainly distributed in the Provinces of Xinjiang, Gansu and Inner Mongolia of Northwest China [10]. It has been used for a long time in desert areas as a high yield forage with good nutritional properties. The young leaves and flowers are also edible and favored by indigenous people [11]. Phenolic compounds, which are usually abundant in leguminous plants [12,13,14], have shown multi-beneficial bioactivities such as antioxidant, anticarcinogenic, antimicrobial, antimutagenic, anti-inflammatory, antiallergic and anti-obesity properties. How to effectively develop those natural phenolic compounds has received much attention. To the best of our knowledge, there are no reports on the chemical composition of *H. halodendron* except for a few phylogenic and cytological studies [15,16]. The aim of this investigation was to develop an efficient method to isolate and purify the main phenolic compounds from *H. halodendron* by HSCCC.

## 2. Results and Discussion

### 2.1. HPLC analysis of the crude extract

The crude ethyl acetate extract from *H. halodendron* was first analyzed by HPLC-UV (K-2501 UV detector). The best separation conditions were gained using methanol-water-acetic acid (50:50:0.5, v/v) as the solvent eluted at a flow rate of 1.0 mL/min at the detection wavelength of 254 nm. 

**Figure 1:**
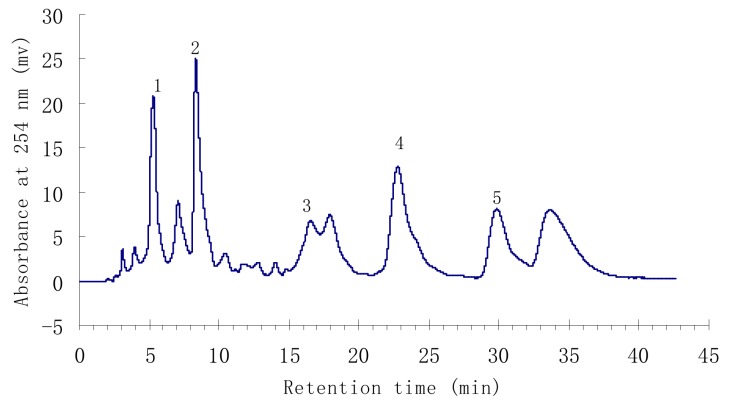
HPLC chromatogram of the ethyl acetate crude extract from *H. halodendron*.

Under the above conditions, a satisfactory separation of the targeted compounds was obtained. The HPLC chromatogram of the ethyl acetate crude extract is shown in Figure 1. Peaks 1 to 5 correspond to *p*-hydroxybenzoic acid (**1**), isorhamnetin-3-*O*-β-D-rutinoside (**2**), quercetin (**3**), 3-*O*-methylquercetin (**4**), and 3,3′-dimethoxy-quercetin (**5**), respectively.

### 2.2. Selection of suitable two-phase solvent system for HSCCC

Successful separation by HSCCC largely depends upon the selection of a suitable two-phase solvent system, which needs an ideal range of the partition coefficient (K) for each target compound [3]. In our test, different solvent systems containing chloroform-methanol-water-acetic acid have been examined to optimize the K values of the five phenolic compounds by HPLC analysis. Their K values were shown in Table 1. The most appropriate K values were obtained at the volume ratio of 4:3:2:0.05 (v/v), which was selected to further isolate and purify the target compounds by HSCCC (TBD-2000 UV detector) in the present study.

### 2.3. Separation of phenolic compounds by HSCCC and chemical structural identification

**Figure 2:**
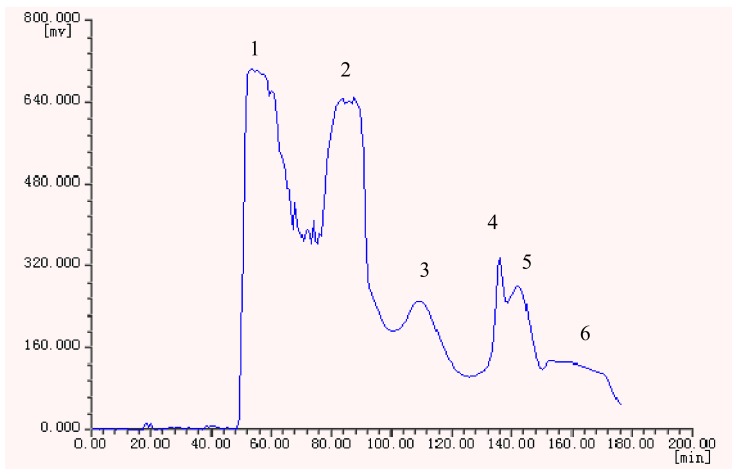
HSCCC chromatogram of the ethyl acetate crude extract from *H. halodendron*.

Under the optimized conditions, six peak fractions were obtained in one-step elution over more than 3 h. The HSCCC chromatogram is shown in Figure 2. 

**Figure 3:**
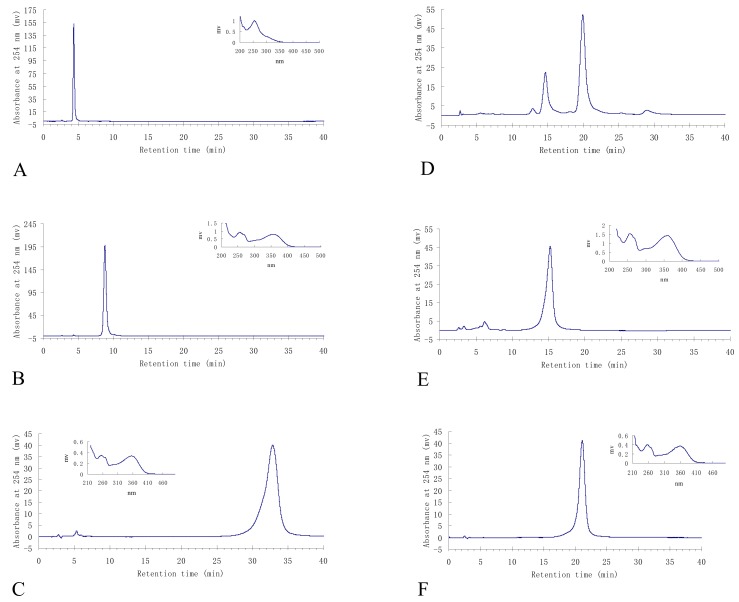
HPLC analyses and UV spectra of the HSCCC fractions. A: *p*-hydroxybenzoic acid (**1**) from HSCCC peak fraction 5; B: isorhamnetin-3-*O*-β-D-rutinoside (**2**) from HSCCC peak fraction 6; C: 3,3′-di-*O*-methylquercetin (**5**) from HSCCC peak fraction 3; D: the mixture of quercetin (**3**) and 3-*O*-methylquercetin (**4**) from HSCCC peak fraction 2; E: quercetin (**3**) separated from the above mixture by preparative HPLC; F: 3-*O*-methylquercetin (**4**) separated from the above mixture by preparative HPLC.

The retention ratio of the ethyl acetate crude extract in the stationary phase was 77.89%. The HSCCC fractions were concentrated and further analyzed by HPLC which gave the chromatograms shown in Figure 3. 

**Figure 4:**
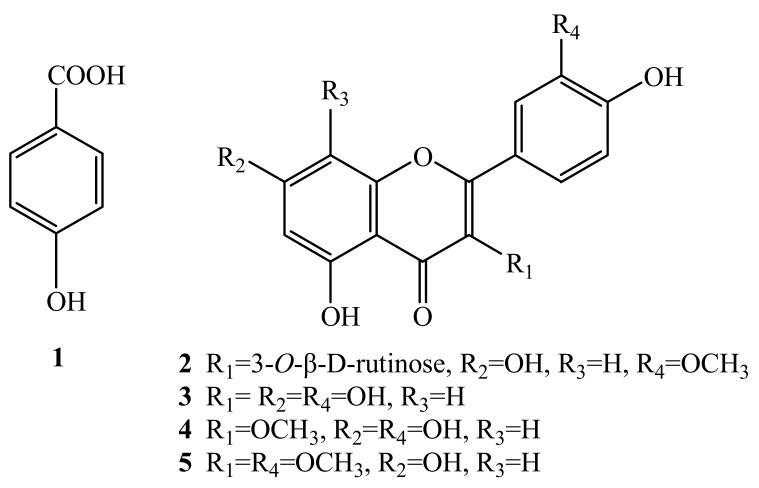
The structures of compounds **1**-**5.**

The HSCCC separation produced 12 mg of compound **2** of 97.99% purity in peak fraction 6, 15 mg of compound **1** at 92.83% of purity in peak fraction 5, 14 mg of **5** at 95.14% of purity in peak fraction 3, and 80 mg of a mixture of **3** and **4** accounted for 26.43% and 71.89% of purity respectively in peak fraction 2. The phenolic compounds prepared by HSCCC were further separated and purified by preparative HPLC and Sephadex LH-20 chromatography. After comparing their physicochemical and spectrometric data with those reported in the literature [17,18,19,20,21], they were identified as *p*-hydroxy-benzoic acid (**1**), isorhamnetin-3-*O*-β-D-rutinoside (**2**), quercetin (**3**), 3-*O*-methylquercetin (**4**), and 3,3′-di-*O*-methylquercetin (**5**), whose structures were shown in Figure 4.

## 3. Experimental

### 3.1. General

Preparative HSCCC was carried out with a model TBE-300B (Tauto Biotech, China) instrument. The apparatus was equipped with a polytetrafluoroethylene tube (diameter of tube was 2.6 mm, and total volume was 280 mL) composed of three preparative coils and a 20-mL sample loop. The HSCCC system was equipped with a TBP-5002 pump and TBD-2000 UV detector operating at 254 nm (Tauto Biotech, China), and a WH500-USB workstation (Wuhao, China). The experimental temperature was 25 °C adjusted by HX-1050 constant temperature circulating implement (Boyikang, China). All organic solvents used for HSCCC were of analytical grade and bought from Beijing Chemical Company. The preparative HPLC system consisted of K-501 pump, K-2501 UV detector (Knauer, Germany), a 2-mL sample loop, a workstation (Lumtech, China), and an Ultimate XB reversed-phase C_18_ column (21.2 mm × 250 mm, 5 μm, Welch Materials, Inc., USA). The analytical HPLC system was similar to that of the preparative system only the 20-μL sample loop and Luna reversed-phase C_18_ column (4.6 mm × 250 mm, 5 μm, Phenomenex, Torrance, USA) were different. Melting points of the compounds were determined on an XT4-100B microscopic melting-point apparatus (Tianjin Tianguang Optical Instruments Company, China) and uncorrected. NMR spectra were recorded on a Bruker-ARX-300 (^1^H at 300 MHz and ^13^C at 75 MHz) or Bruker Avance DRX-500 (^1^H at 500 MHz and ^13^C at 125 MHz) spectrometers. ESI-MS spectra were recorded on a Bruker Esquire 6000 LC/MS spectrometer. Methanol used for HPLC analysis was of chromatographic grade and purchased from Tianjin Tianhao Chemical Company.

### 3.2. Plant material

The aerial parts of *H. halodendron* were collected in June 2008 at Shihezi of Xinjiang Province of China, and authenticated by Professor Pin Yan of Shihezi University of Xinjiang. A voucher specimen of this collection (BSMPMI-200806002) was deposited at the Herbarium of the Institute of Chinese Medicinal Materials, China Agricultural University. The plant materials were left to dry in the shade at room temperature to a constant weight.

### 3.3. Preparation of the crude extract

The air-dried and powdered aerial parts (1 kg) of *H. halodendron* were soaked three times in 95% ethanol (5 L) at room temperature for an interval of 10 days. After the combined filtrate was concentrated under vacuum at 50 °C, the brown residue (125 g) was suspended in water. It was extracted with petroleum ether, and then with ethyl acetate. The ethyl acetate fraction was concentrated under vacuum at 50 °C to obtain 5 g of the extract, which was used for HSCCC separation.

### 3.4. Selection of the two-phase solvent system

The composition of the two-phase solvent system (Table 1) was selected according to the partition coefficient (K value) of the target compounds. The K-values were determined by HPLC as follows: approximately 0.2 mg of crude sample was added to a test tube to which 3.0 mL of the lower phase of the pre-equilibrated two-phase solvent system was added. After the crude sample thoroughly dissolved, equal volume of the upper phase of the pre-equilibrated two-phase solvent system was added and shaken violently for several minutes. Finally, the upper and lower phases were analyzed by HPLC. The K values of all components in sample were calculated according to the ratio of the peak areas. K = A_U_/A_L_, where A_U_ was the peak area of the upper phase, and A_L_, the peak area of the lower phase.

### 3.5. Preparation of the two-phase solvent systems and sample solution

The selected solvent system, chloroform-methanol-water-acetic acid (4:3:2:0.05, v/v), was prepared by adding all the solvents to a separation funnel according to the volume ratios and thoroughly equilibrated by shaking repeatedly. After thoroughly equilibrated, the upper phase and lower phase were separated and degassed by sonication for 30 min prior to use. The sample solution was prepared by dissolving the crude sample (700 mg) in 20 mL of the mixture of equal volume of lower phase and upper phase of the solvent system used for HSCCC separation, and sonicated for several minutes before loading into the column.

### 3.6. HSCCC separation procedure

The coil column was first entirely filled with the stationary phase (the upper phase) of the solvent system. Then the apparatus was rotated at 850 rpm, while the mobile phase (the lower phase) was pumped into the column at a flow rate of 3 mL/min. After the mobile phase front emerged, and hydrodynamic equilibrium was established in the column, about 20 mL of sample solution containing 700 mg of the ethyl acetate extract was injected through the injection valve. The effluent of the column was continuously monitored with a UV detector at 254 nm. Peak fractions were collected according to the elution profile. The temperature of the apparatus was set at 25 °C. The yield was 80 mg (yield 11.4% of the ethyl acetate extract) for peak fraction 2, 14 mg (yield 2.0%) for peak fraction 3, 15 mg (yield 2.1%) for peak fraction 5, and 12 mg (yield 1.7%) for peak fraction 6. Both fractions 1 (54 mg, yield 7.7%) and 4 (136 mg, yield 19.4%) were examined by TLC to be complicated with many minor compounds.

### 3.7. Analysis and identification of HSCCC peak fractions

The ethyl acetate crude extract and peak fractions separated by HSCCC were analyzed by HPLC. The analyses were performed with a reversed-phase C_18_ column at 25 °C. The mobile phase composed of methanol-water-acetic acid (50:50:0.5, v/v) was eluted at a flow rate of 1.0 mL/min and the effluent monitored at 254 nm. The mixture of the peak fraction 2 from HSCCC was further separated by preparative HPLC with a reversed-phase C_18_ column eluted at a flow rate of 10.0 mL/min, other conditions were the same as above. The separated compounds were further purified by Sephadex LH-20 with chloroform-methanol (1:1, v/v) as the elution system. The linear equations of the compounds by HPLC analysis were as follows: *Y* = 14.5850*X* + 6.6032 (for *p*-hydroxybenzoic acid, **1**), *Y* = 13.7380*X* - 137.9600 (for isorhamnetin-3-*O*-β-D-rutinoside, **2**), *Y* = 16.5840*X* - 93.7360 (for quercetin, **3**), *Y* = 22.4450*X* + 256.9700 (for 3-*O*-methylquercetin, **4**), *Y* = 59.5140*X* - 34.3180 (for 3,3′-di-*O*-methylquercetin, **5**), where *Y* was the peak area, and *X* was the sample concentration (μg/mL). The physicochemical and spectrometric data of five phenolic compounds were given as follows.

*p-Hydroxybenzoic acid* (**1**). white amorphous powder (MeOH); m.p. 210-213 °C; UV λ_max_ (MeOH) nm: 204, 252; ^1^H-NMR (acetone-*d_6_*, 500 MHz) δ (ppm), 7.92 (2H, d, *J* = 9.0 Hz, H-2 and H-6), 6.92 (2H, d, *J* = 6.92 Hz, H-3 and H-5). The structure was confirmed by comparison with literature data [17], 

*Isorhamentin-3-O-β-D-rutinoside* (**2**). Yellow amorphous powder, m.p. 186-187 °C. UV λ_max_ (MeOH) nm: 255, 355. ESI-MS *m*/*z* 623 [M-H]^-^, 647 [M+Na]^+^; ^1^H-NMR (DMSO-*d_6_*, 300 MHz) δ (ppm), 12.50 (1H, s, OH-5), 6.19 (1H, d, *J* = 1.9 Hz, H-6), 10.89 (1H, s, OH-7), 6.41 (1H, d, *J* = 1.8 Hz, H-8), 7.86 (1H, d, *J* = 2.0 Hz, H-2′), 3.83 (3H, s, OCH_3_-3′), 9.83 (1H, s, OH-4′), 6.92 (1H, d, *J* = 8.4 Hz, H-5′), 7.53 (1H, dd, *J* = 2.0, 8.4 Hz, H-6′), 5.44 (1H, d, *J* = 7.3 Hz, glc-H-1), 4.41 (1H, d, *J* = 1.4 Hz, rha-H-1), 0.98 (1H, d, *J* = 5.6 Hz, rha-H-6); ^13^C-NMR (DMSO-*d_6_*, 75 MHz) δ (ppm), 156.7 (C-2), 133.2 (C-3), 177.4 (C-4), 161.4 (C-5), 99.1 (C-6), 165.0 (C-7), 94.1 (C-8), 156.5 (C-9), 103.9 (C-10), 121.2 (C-1′), 113.5 (C-2′), 149.6 (C-3′), 55.9 (OCH_3_-3′), 147.1 (C-4′), 115.5 (C-5′), 122.5 (C-6′), 101.5 (glc-1), 74.5 (glc-2), 76.6 (glc-3), 70.8 (glc-4), 76.1 (glc-5), 67.0 (glc-6), 101.1 (rha-1), 70.5 (rha-2), 70.8 (rha-3), 72.0 (rha-4), 68.5 (rha-5), 17.8 (rha-6). The structure was confirmed by comparison with literature data [18].

*Quercetin* (**3**). Yellow amorphous powder (MeOH); m.p. 314-315 °C; UV λ_max_ (MeOH) nm: 255, 372; ESI-MS *m*/*z* 303 [M+H]^+^, 301 [M-H]^-^; ^1^H-NMR (DMSO-*d_6_*, 300 MHz) δ (ppm), 9.38 (1H, s, OH-3), 12.49 (1H, s, OH-5), 6.19 (1H, d, *J* = 2.0 Hz, H-6), 10.77 (1H, s, OH-7), 6.42 (1H, d, *J* = 2.0 Hz, H-8), 7.68 (1H, d, *J* = 2.2 Hz, H-2′), 9.38 (1H, s, OH-3′), 9.38 (1H, s, OH-4′), 6.90 (1H, d, *J* = 8.5 Hz, H-5′), 7.56 (1H, dd, *J* = 2.2, 8.5 Hz, H-6′); ^13^C-NMR (DMSO-*d_6_*, 125 MHz) δ (ppm), 147.9 (C-2), 135.8 (C-3), 176.0 (C-4), 160.8 (C-5), 98.3 (C-6), 164.1 (C-7), 93.5 (C-8), 156.3 (C-9), 103.1 (C-10), 122.1 (C-1′), 115.2 (C-2′), 145.2 (C-3′), 146.9 (C-4′), 115.7 (C-5′), 120.1 (C-6′). The structure was confirmed by comparison with literature data [19].

*3-O-Methylquercetin* (**4**). Yellow and needle crystal (MeOH); m.p. 256-257 °C; UV λ_max_ (MeOH) nm: 256, 358; ESI-MS *m*/*z* 315 [M-H]^-^; ^1^H-NMR (DMSO-*d_6_*, 300 MHz) δ (ppm), 3.78 (3H, s, OCH_3_-3), 12.71 (1H, s, OH-5), 6.20 (1H, d, *J* = 2.0 Hz, H-6), 10.82 (1H, s, OH-7), 6.41 (1H, d, *J* = 2.1 Hz, H-8), 7.56 (1H, d, *J* = 2.2 Hz, H-2′), 10.82 (1H, s, OH-3′), 10.82 (1H, s, OH-4′), 6.92 (1H, d, *J* = 8.5 Hz, H-5′), 7.40 (1H, dd, *J* = 2.3, 8.4 Hz, H-6′); ^13^C-NMR (DMSO-*d_6_*, 75 MHz) δ (ppm), 155.8 (C-2), 137.8 (C-3), 178.0 (C-4), 161.4 (C-5), 93.7 (C-6), 164.3 (C-7), 98.7 (C-8), 156.5 (C-9), 104.3 (C-10), 121.0 (C-1′), 115.6 (C-2′), 145.4 (C-3′) , 59.8 (OCH_3_-3′), 148.9 (C-4′), 115.9 (C-5′), 120.7 (C-6′). The structure was confirmed by comparison with literature data [20].

*3,3′-di-O-Methylquercetin* (**5**). Yellow and needle crystal (chloroform); m.p. 210-212 °C; UV λ_max_ (MeOH) nm: 268, 360; ESI-MS *m*/*z* 329 [M-H]^-^, 353 [M+Na]^+^; ^1^H-NMR (DMSO-*d_6_*, 300 MHz) δ (ppm), 3.80 (3H, s, OCH_3_-3), 12.68 (1H, s, OH-5), 6.20 (1H, d, *J* = 2.0 Hz, H-6), 10.82 (1H, s, OH-7), 6.48 (1H, d, *J* = 2.1 Hz, H-8), 7.64 (1H, d, *J* = 2.1 Hz, H-2′), 3.86 (1H, s, OCH_3_-3′), 9.88 (1H, s, OH-4′), 6.97 (1H, d, *J* = 8.4 Hz, H-5′), 7.58 (1H, dd, *J* = 2.1, 8.4 Hz, H-6′); ^13^C-NMR (DMSO-*d_6_*, 125 MHz) δ (ppm), 155.6 (C-2), 137.9 (C-3), 59.9 (OCH_3_-3), 178.1 (C-4), 161.4 (C-5), 94.0 (C-6), 164.3 (C-7), 98.8 (C-8), 156.5 (C-9), 104.4 (C-10), 122.4 (C-1′), 112.3 (C-2′), 150.0 (C-3′), 55.9 (OCH_3_-3′), 147.6 (C-4′), 115.8 (C-5′), 121.0 (C-6′). The structure was confirmed by comparison with literature data [21].

## 4. Conclusions

Our study demonstrates that HSCCC is an effective and rapid method in separating phenolic compounds from natural plant resources. Using HSCCC, three phenolic compounds including *p*-hydroxybenzoic acid (**1**), isorhamnetin-3-*O*-β-D-rutinoside (**2**), and 3,3′-di-*O*-methylquercetin (**5**), along with a phenolic mixture containing quercetin **(3**) and 3-*O*-methylquercetin (**4**) were successfully separated from *H. halodendron* with a chloroform-methanol-water-acetic acid (4:3:2:0.05, v/v) two-phase solvent system. The present study will lay a foundation for a large scale preparation of the phenolic compounds from the aerial parts of *H. halodendron*. More research work should be devoted to evaluate biological activities (*i.e*. antioxidant and antimicrobial properties) of these phenolic compounds that may be applied in food, agriculture and medicine industry as a source of biologically active agents. Furthermore, screening of an alternative more environmentally friendly two-phase solvent system without chloroform is also necessary.

## Figures and Tables

**Table 1:** The partition coefficients (K values) of the phenolic compounds with chloroform-methanol-water-acetic acid as the two-phase solvent system by HPLC analysis.

No.	Ratio (v/v)	K value				
		Peak 1	Peak 2	Peak 3	Peak 4	Peak 5
1	3:3:2:0.05	0.22	0.61	0.84	2.32	0.52
2	4:3:2:0.05	1.95	1.11	1.28	1.41	0.63
3	4:4:2:0.05	0.90	0.63	0.90	1.43	0.57
4	4:2:2:0.05	0.48	0.90	0.79	4.70	0.53
5	5:3:2:0.05	0.27	0.35	0.85	1.26	0.28

"Ratio" is expressed as the volume ratio of chloroform-methanol-water-acetic acid. Peaks 1-5 corresponded to compounds **1**-**5**, respectively.
